# Association of Retinal Microvascular Characteristics With Short-term Memory Performance in Children Aged 4 to 5 Years

**DOI:** 10.1001/jamanetworkopen.2020.11537

**Published:** 2020-07-24

**Authors:** Leen J. Luyten, Yinthe Dockx, Narjes Madhloum, Hanne Sleurs, Nele Gerrits, Bram G. Janssen, Kristof Y. Neven, Michelle Plusquin, Eline B. Provost, Patrick De Boever, Tim S. Nawrot

**Affiliations:** 1 Centre for Environmental Sciences, Hasselt University, Hasselt, Belgium; 2 Unité de Recherche en Biologie Cellulaire–Namur Research Institute for Life Sciences, Namur University, Namur, Belgium; 3 Health Unit, Flemish Institute for Technological Research, Mol, Belgium; 4 Department of Public Health and Primary Care, Occupational and Environmental Medicine, Leuven University, Leuven, Belgium

## Abstract

**Importance:**

Neurocognitive functions develop rapidly in early childhood and depend on the intrinsic cooperation between cerebral structures and the circulatory system. The retinal microvasculature can be regarded as a mirror image of the cerebrovascular circulation.

**Objective:**

To investigate the association between retinal vessel characteristics and neurological functioning in children aged 4 to 5 years.

**Design, Setting, and Participants:**

In this cohort study, mother-child pairs were recruited at birth from February 10, 2010, to June 24, 2014, and renewed consent at their follow-up visit from December 10, 2014, to July 13, 2018. Participants were followed up longitudinally within the prospective Environmental Influence on Aging in Early Life birth cohort. A total of 251 children underwent assessment for this study. Data were analyzed from July 17 to October 30, 2019.

**Main Outcomes and Measures:**

Retinal vascular diameters, the central retinal arteriolar equivalent (CRAE), central retinal venular equivalent (CRVE), vessel tortuosity, and fractal dimensions were determined. Attention and psychomotor speed, visuospatial working memory, and short-term visual recognition memory were assessed by the Cambridge Neuropsychological Test Automated Battery, including the following tasks: Motor Screening (MOT), Big/Little Circle (BLC), Spatial Span (SSP), and Delayed Matching to Sample (DMS).

**Results:**

Among the 251 children included in the assessment (135 girls [53.8%]; mean [SD] age, 4.5 [0.4] years), for every 1-SD widening in CRVE, the children performed relatively 2.74% (95% CI, −0.12 to 5.49; *P* = .06) slower on the MOT test, had 1.76% (95% CI, −3.53% to −0.04%; *P* = .04) fewer correct DMS assessments in total, and made 2.94% (95% CI, 0.39 to 5.29; *P* = .02) more errors given a previous correct answer in the DMS task on multiple linear regression modeling. For every 1-SD widening in CRAE, the total percentage of errors and errors given previous correct answers in the DMS task increased 1.44% (95% CI, −3.25% to 0.29%; *P* = .09) and 2.30% (95% CI, −0.14% to 4.61%; *P* = .07), respectively. A 1-SD higher vessel tortuosity showed a 4.32% relative increase in latency in DMS task performance (95% CI, −0.48% to 9.12%; *P* = .07). Retinal vessel characteristics were not associated with BLC and SSP test outcomes.

**Conclusions and Relevance:**

These findings suggest that children’s microvascular phenotypes are associated with short-term memory and that changes in the retinal microvasculature may reflect neurological development during early childhood.

## Key Points

**Question:**

Do retinal microvascular traits reflect neurocognitive development in early childhood?

**Findings:**

In this cohort study of 251 children aged 4 to 5 years, retinal venular widening and a higher vessel tortuosity were associated with a lower performance of short-term visual recognition memory, assessed by means of the Delayed Matching to Sample task of the Cambridge Neuropsychological Test Automated Battery.

**Meaning:**

These associations between retinal microvascular characteristics and neurocognitive development in young children provide fundamental information on the association between the microvasculature and neurocognition from early life onwards.

## Teaser

This cohort study investigates the association between retinal vessel characteristics and neurological functioning in children aged 4 to 5 years.

## Introduction

Neurological development is at its most crucial phase during early childhood. From early gestation, important brain structures are formed and reshaped, and dendritic connections of neuronal networks are constantly established until about 5 years of age.^1^ Cognitive performance depends on properly functioning cerebral blood circulation, because this part of the vascular network entails approximately 15% of the total human cardiovascular output.^2^ Various studies have shown that failure to maintain good cardiovascular health from a young age onward results in a lower scoring of numerous neurological outcomes later in life, such as psychomotor speed, executive function, and verbal memory.^3,4^

The microcirculation is a large part of the human vasculature that undergoes extensive, organ-specific perinatal maturation.^5,6^ The retina is an outgrowth of the developing brain, and both tissues share morphological and functional characteristics. Therefore, the retinal microvasculature can be considered as a proxy of the conditions of the blood vessels in the brain.^7^ Structural changes in the retinal vasculature can be early markers for the development of cerebral vascular disease.^8,9^ In previous research,^10^ smaller retinal arteriolar calibers, summarized as the central retinal arteriolar equivalent (CRAE), have been linked to a lower IQ score in children aged 11 years. However, to date, retinal vessel diameters and other metrics, such as tortuosity, have never been studied in association with neurocognitive functioning in children as young as 4 years.

We have studied the association between retinal microvascular characteristics and neurocognitive functioning in children aged 4 to 5 years in the prospective Environmental Influence on Aging in Early Life (ENVIRONAGE) birth cohort. By means of the Cambridge Neuropsychological Test Automated Battery (CANTAB), we investigated the association between the retinal vasculature and psychomotor speed, visuospatial short-term working memory, and visual short-term recognition memory.

## Methods

### Study Population

Mother-child pairs participating in this study were recruited as part of the ongoing prospective ENVIRONAGE birth cohort. Study protocols of the recruitment phase at birth (from February 10, 2010, to June 24, 2014) and the follow-up phase at 4 years of age were approved by the ethics committees of both the Hospital East-Limburg, Genk, Belgium, and Hasselt University, Diepenbeek, Belgium. Details on recruitment of eligible mother-child pairs is described elsewhere.^11^ Mothers and their children were reinvited to participate in the first follow-up visit of this prospective cohort study when the child was aged 4 to 6 years. Written consent was obtained from all participating mothers, and children gave their oral assent. Follow-up examinations took place from December 10, 2014, to July 13, 2018. Within this timeframe, 587 children were aged 4 to 6 years and could participate in this phase of the study. Thirteen mother-child pairs were not eligible for participation because the child had died (n = 1) or they had moved abroad or too far from our examination center (n = 12). Of the remaining 574 mother-child pairs, 74 women could not be contacted because no up-to-date contact details could be retrieved, 3 mother-child pairs could not be contacted within the timeframe when the child was 4 to 6 years of age, 165 women refused to participate, and 332 renewed consent. Hence, this resulted in a final participation rate of 57.8%. This study followed the Strengthening the Reporting of Observational Studies in Epidemiology (STROBE) reporting guideline.

Of the 332 participating mother-child pairs, 74 were not included in the statistical analyses because no good-quality images of the retinal vasculature of either eye were available. Pictures could not be obtained in 24 participants owing to a medical condition (n = 4) or a lack of concentration or unwillingness to participate (n = 20). For 50 participants, the quality of the pictures was suboptimal, owing to children’s movement of their body or eyes during the capturing of the images. Finally, for 7 additional participants, no CANTAB data could be obtained, resulting in a study population of 251 participants (eFigure in the Supplement).

At the follow-up visit, the mothers filled out several questionnaires to obtain general information on the lifestyle of the child and the parents, the emotional well-being of the mother at the moment of the follow-up examinations, and the emotions and behavior of their child, summarized during the last 6 months before the follow-up examination via the Strengths and Difficulties Questionnaire, which has been validated in other cohorts studying children in this age group.^12^ For the Strengths and Difficulties Questionnaire, the first 25 of a total of 33 questions are subdivided into 5 subscales: Hyperactivity, Emotional Symptoms, Conduct Problems, Peer Problems, and Prosocial Behavior. The scores of the first 4 subscales were summarized into a problem score, with a final score ranging from 0 to 40 and higher scores indicating a more problematic emotional behavior.

### Clinical Measurements

A single trained examiner performed the measurements of the clinical parameters (height, weight, and blood pressure) (N.M.), obtained the fundus pictures (L.J.L.), and gave the instructions for the administration of the CANTAB (L.J.L.). It was made clear at the beginning of the clinical examinations that each test could be stopped at any moment if the child was uncomfortable or scared. The participant’s blood pressure was measured with an automated upper-arm blood-pressure monitor (Omron 705IT; Omron Corporation), equipped with a cuff adapted to the arm size of children. Measurements were performed by a standardized method, as described by the European Society of Hypertension.^13^ In summary, after 5 minutes of rest in supine position, a trained observer (N.M.) obtained 5 consecutive readings of the systolic blood pressure (SBP) and diastolic blood pressure (DBP) with 1-minute intervals. Mean SBP and DBP were based on the mean of the last 3 readings. Mean arterial pressure (MAP) was calculated via the following equation: MAP = (2/3 × DBP) + (1/3 × SBP).

To determine the retinal blood vessel parameters, fundus pictures of the right and left eyes were obtained with a 45° 6.3-megapixel digital nonmydriatic retinal camera (Canon CR-2 plus; Hospithera NV). These pictures were subsequently analyzed with the MONA-REVA software, version 2.1.1 (VITO Health). First, the diameter of the optic disc was determined for each picture, and all distance measurements within the fundus were set relative to this value. Next, the widths of the retinal arterioles and venules were calculated in the zone 0.5 to 1.0 times the diameter of the optic disc. The diameters of the 6 largest arterioles and 6 largest venules in this zone were used in the revised Parr-Hubbard formula to calculate the CRAE and central retinal venular equivalent (CRVE).^14^ The tortuosity index is computed as the mean tortuosity of the branch segments, where the tortuosity of a branch segment is the ratio of the line traced on each tree along the vessel axis from 0.5 to 2.0 times the optic disc diameter and the line connecting the end points. Segmentations are cropped centered on the optic disc whereby the inner and outer radii were taken at 1.5 and 5.0 times the radius of the optic disc.^15^ Finally, multifractal analysis was performed in the FracLac plugin in ImageJ (Laboratory for Optical and Computational Instrumentation, University of Wisconsin) using the segmentations computed by MONA-REVA. The applied segmentation algorithm used multiscale line filtering based on the method of Nguyen et al^15^ and postprocessing steps, such as double thresholding, blob extraction, removal of small connected regions, and filling holes. Settings of the ImageJ fractal dimension extraction in the FracLac plugin were the box-counting method, 10 grid positions, scaled series for calculating grid calibers, and Q range of −10, +10, and 0.1. We calculated the generalized dimensions (D_q_) for q = 0, 1, and 2, which are the capacity dimension (D_0_), the information dimension (D_1_), and correlation dimension (D_2_), the curve asymmetry, and the singularity length (Δα). For a multifractal structure, the following applies: D_0_≥D_1_≥D_2_.^16^

When both fundus images of the child were available (n = 201), the mean values of the CRAE, CRVE, and tortuosity index were used in the analyses. For 29 individuals, the calculations were available for the left eye only, whereas for 21 children, the data of the right eye only were used for analysis. For these 50 individuals, the fundus image of the other eye was unavailable or of insufficient quality. No difference could be determined between the retinal arteriolar diameter, retinal venular diameter, or vessel tortuosity of either 1 or 2 eyes.

### CANTAB Tasks

**Figure 1.**
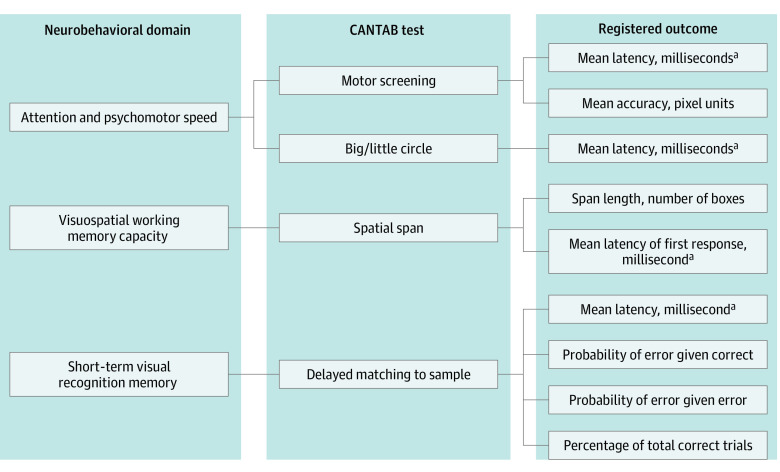
Schematic Representation of the Clinical Meaurements Used in this Study The 4 Cambridge Neuropsychological Test Automated Battery (CANTAB) tasks are shown with the corresponding neurobehavioral domains that are assessed with these tests and the specific outcomes that are registered for each of the tasks. ^a^Latency has an opposite association with reaction speed: a slower reaction time equals an increment of the latency value.

The neurocognitive development of the children was assessed by means of 4 tasks (Figure 1) of the CANTAB (University of Cambridge), performed on a touch screen tablet. All instructions necessary to understand and complete the tasks were given according to a standardized script provided by the software developers. The examiner always made sure that the child used only the index finger of their dominant hand. If the child was unsure about whether he or she was performing the test correctly or if the child did not understand the task, the task description was repeated once during the test. Participants were encouraged when they were completing the test correctly.

The first CANTAB task we used was Motor Screening Task (MOT), which familiarizes the child with the touch screen and verifies whether oral descriptions given by the examiner are well understood. By touching a series of crosses on the screen as fast as possible, MOT assesses the reaction speed (latency in milliseconds) and the mean error, which measures the accuracy of touch in pixel units between the point of touch and the center of the target (based on a screen resolution of 640 × 350 pixels). Next, the child executes the Big/Little Circle (BLC) task. Similar to the MOT, this task familiarizes the participant with oral task descriptions and a touch screen. The child must first select the smallest of 2 circles. After a series of 20 assessments, the child has to touch the biggest circle for another series of 20 tests. The outcome measures of the BLC task covers the speed of response (latency in milliseconds) as well as the number of correct answers. Both the MOT and the BLC task perform an assessment of attention and psychomotor speed.

Subsequently, the child performed the Spatial Span (SSP) task to assess short-term memory. Nine white squares were shown on the screen, of which initially 2 boxes changed color in a certain sequence. The child then had to touch the boxes in the same order as they changed color. When the task was performed correctly, an additional box changed color in the next sequence, for a series of as many as 9 boxes. The sequences varied throughout the test. The child could try again if he or she chose the wrong sequence or square, for a maximum of 3 times. After 3 subsequent errors, the test was ended. Outcome measures for the SSP task were given as the maximum number of boxes that the child could remember in the correct sequence (span length) and the time to initiate touching the sequence (mean latency to first answer in milliseconds). Finally, the Delayed Matching to Sample (DMS) task was performed, which measured visual matching ability and visual recognition memory. In the center of the screen, a complex visual pattern was shown. After several seconds, the child had to choose this pattern from 4 similar patterns. These choice figures would either appear on the screen simultaneously with the first (sample) figure or with a delay of either 4 or 12 seconds. Reported outcomes were the percentage of correct answers (all assignments or only those at which the choice patterns are shown with a delay) and the latency from when the choice patterns were displayed until choosing the correct pattern, if the first touched choice was the correct one.

### Statistical Analysis

Data were analyzed from July 17 to October 30, 2019. We used SAS software, version 9.4 (SAS Institute, Inc) for data analyses. Correlations between the retinal vessel characteristics were assessed by Pearson correlation. We tested differences between the retinal arteriolar and venular diameter, retinal vessel curvature of either 1 or 2 eyes, and microvascular and CANTAB outcomes between boys and girls with a 2-sided *t* test. The latency data of MOT, BLC, SSP, and DMS were log-transformed to better comply with linear model assumptions. Other test outcomes were studied without additional transformation. We combined CANTAB outcomes assessing the same neurological area into 2 different principal components using the PROC PINCOMP procedure. We then used separate multivariable linear regression models to estimate associations between retinal vessel characteristics and cognitive functioning, using the first 2 principal components as the dependent variables. These first 2 principal components combined explained more than 50% of the data variance. Subsequently, we tested the associations between retinal vessel characteristics and all separate outcome variables of the 4 CANTAB tasks in the battery by means of multivariable linear regression models. All models were adjusted for the child’s sex, age, body mass index (BMI; calculated as weight in kilograms divided by height in meters squared), gestational age, birth weight, and ethnicity. In a subsequent analysis of the models assessing the separate test outcomes, we additionally adjusted for the MAP, the season in which the follow-up examination took place, the time of day at which the CANTAB task was performed, and the problem score as a representation of emotional state and behavior. The magnitude of the association was expressed for every 1-SD increment in retinal vessel characteristic.

We performed 4 additional sensitivity analyses. In the first sensitivity analysis, we additionally adjusted the model for the educational level of both mother and father at the moment of the follow-up examination, the smoking habits and alcohol use of the mother during pregnancy, and the exposure of the child to smoking by the parents. Second, a sensitivity analysis was conducted to assess the association between retinal vessel characteristics and CANTAB task outcomes in a population excluding mothers who consumed alcohol during pregnancy (n = 50), because prenatal exposure to alcohol is known to have substantial effects on neurological development of the fetus, which can also be reflected by changes in retinal vessel tortuosity and various other biomarkers.^17^ A third sensitivity analysis was performed in the subset of children excluding those with a high problem score assessed with the Strengths and Difficulties Questionnaire (ie, 14 to 40 points) (n = 33). A final sensitivity analysis was conducted, excluding children who were born prematurely (ie, gestational age of ≤37 weeks) (n = 11). Two-sided *P* < .05 indicated significance.

## Results

### Study Population Characteristics

The study characteristics of the 251 participating mother-child pairs are provided in Table 1. On the day of the measurements, the children (135 girls [53.8%] and 116 boys [46.2%]) had a mean (SD) age of 4.5 (0.4) years and MAP of 68.6 (6.0) mm Hg. Mean (SD) BMI was 16.0 (1.5). Examinations mostly took place during spring (82 [32.7%]) and winter (77 [30.7%]). In their home environment, 39 children (15.5%) were exposed to passive smoking via 1 parent and 34 (13.5%) via both parents.

**Table 1.** Characteristics of the Mother-Child Pairs Included in This Study

Characteristic	No. of participants (%) (n = 251)^a^
**Child**	
Age at follow-up, mean (SD), y	4.5 (0.4)
Sex	
Male	116 (46.2)
Female	135 (53.8)
Ethnicity	
European	236 (94.0)
Non-European	15 (6.0)
Measurement at follow-up, mean (SD)	
Height, cm	107.7 (5.0)
Weight, kg	18.7 (2.7)
BMI	16.0 (1.5)
Season at follow-up	
Spring	82 (32.7)
Summer	51 (20.3)
Autumn	41 (16.3)
Winter	77 (30.7)
Blood pressure, mean (SD), mm Hg	
Systolic	97.7 (8.2)
Diastolic	54.0 (6.9)
Mean arterial pressure, mm Hg	68.6 (6.0)
Exposure to passive smoking	
Not exposed	166 (66.1)
Exposed via one parent	39 (15.5)
Exposed via both parents	34 (13.5)
Information missing	12 (4.9)
**Mother**	
Age at child’s birth, mean (SD), y	29.9 (4.2)
Prepregnancy BMI, mean (SD)	24.4 (4.7)
Smoking behavior during pregnancy	
Never smoked	174 (69.3)
Stopped smoking before pregnancy	43 (17.1)
Smoked during pregnancy	34 (13.5)
Alcohol consumption during pregnancy	
Yes	50 (19.9)
No	196 (78.1)
Information missing	5 (2.0)
Educational level	
Low (no high school diploma)	14 (5.6)
Middle (high school diploma)	59 (23.5)
High (college degree or higher)	178 (70.9)
**Father**	
Educational level	
Low (no high school diploma)	16 (6.4)
Middle (high school diploma)	106 (42.2)
High (college degree or higher)	118 (47.0)
Information missing	11 (4.4)

Abbreviation: BMI, body mass index (calculated as weight in kilograms divided by height in meters squared).

^a^ Unless otherwise indicated, data are expressed as number (percentage) of participants. Percentages have been rounded and may not total 100.

Mothers gave birth at a mean (SD) age of 29.9 (4.2) years. Most of the mothers did not consume alcohol (196 [78.1%]) and had never smoked (174 [69.3%]) throughout their pregnancy. Most mothers (178 [70.9%]) had a college education or higher, as did fewer fathers (118 [47.0%]) (Table 1).

### Microvasculature Characteristics

For the entire study population, the mean (SD) measurement of the retinal arteriolar and venular diameter was 180.9 (14.4) μm and 251.1 (19.6) μm, respectively, and the mean (SD) vessel tortuosity value was 0.889 (0.012) (Table 2). These 3 retinal vessel characteristics were slightly increased in girls compared with boys, with a small but statistically significant difference for the CRVE (253.4 [18.1] vs 248.3 [20.9] μm; *P* = .04). The multifractal outcomes of the study population were similar to those of the boys and girls separately, with 1.56 (0.03) for D_0_ (capacity dimension), 1.52 (0.03) for D_1_ (information dimension), 1.51 (0.03) for D_2_ (correlation dimension), 0.27 (0.05) for curve asymmetry, and a singularity length of 0.76 (0.07) for all children combined. A positive correlation was found between both the arteriolar and venular diameter (*r* = 0.58; *P* < .001) and the venular diameter and vessel tortuosity (*r* = 0.18; *P* = .004), and a borderline positive correlation was found between retinal arteriolar diameter and vessel tortuosity (*r* = 0.12; *P* = .06).

**Table 2.** CRAE, CRVE, Retinal Vessel Tortuosity Index, and Multifractal Characteristics of the Study Population

Characteristic	Study Population, mean (SD)			*P* value^a^
	Combined	Girls	Boys	
CRAE, μm	180.9 (14.4)	182.4 (13.6)	179.3 (15.2)	.10
CRVE, μm	251.1 (19.6)	253.4 (18.1)	248.3 (20.9)	.04
Tortuosity index^b^	0.889 (0.012)	0.890 (0.012)	0.887 (0.013)	.07
D_0_	1.56 (0.03)	1.56 (0.03)	1.56 (0.03)	.63
D_1_	1.52 (0.03)	1.52 (0.03)	1.52 (0.04)	.65
D_2_	1.51 (0.03)	1.51 (0.03)	1.50 (0.04)	.66
Curve asymmetry	0.27 (0.05)	0.27 (0.05)	0.27 (0.06)	.69
Singularity length	0.76 (0.07)	0.76 (0.07)	0.76 (0.07)	.77

Abbreviations: CRAE, central retinal arteriolar equivalent; CRVE, central retinal venular equivalent; D_0_, capacity dimension; D_1_, information dimension; D_2_, correlation dimension.

^a^ Calculated using a 2-sided *t* test.

^b^ Computed as the mean tortuosity of the branch segments, where the tortuosity of a branch segment is the ratio of the line traced on each tree along the vessel axis from 0.5 to 2.0 times the optic disc diameter and the line connecting the end points.

### Cognitive Characteristics

CANTAB task outcomes are summarized in Table 3, for all participants and for boys and girls separately. All latency outcomes are expressed as geometric means. There was no significant difference in task performance between boys and girls. The mean time to touch the target of the MOT task was 949.2 (95% CI, 920.7-978.6) milliseconds. Mean accuracy of the MOT task was 13.9 (95% CI, 13.6-14.3) pixel units removed from the center of the target cross. The latency of the BLC task was similar to that of the MOT task: the mean time needed to tap the correct circle was 1075.3 (95% CI, 1050.5-1100.7) milliseconds.

**Table 3.** Outcomes of the 4 Tasks of the CANTAB

Task and outcome(s)	Mean (95% CI)			*P* value^a^
	Combined (n = 251)	Boys (n = 116)	Girls (n = 135)	
MOT				
Latency, ms^b^	949.2 (920.7-978.6)	939.7 (896.8-984.6)	957.4 (919.4-997.0)	.55
Mean accuracy, pixel units	13.9 (13.6-14.3)	13.9 (13.5-14.3)	13.9 (13.4-14.5)	.88
BLC				
Latency, ms^b^	1075.3 (1050.5-1100.7)	1098.9 (1057.0-1142.5)	1055.5 (1026.6-1085.2)	.10
SSP				
Span length, No. of boxes	2.7 (2.6-2.9)	2.7 (2.5-3.0)	2.7 (2.5-2.9)	.93
Latency to first response, ms^b^	3666.3 (3498.2-3842.4)	3621.8 (3374.1-3887.6)	3704.9 (3477.7-3947.0)	.64
DMS				
Latency, ms^b^	4102.2 (3942.5-4268.5)	4201.0 (3936.2-4483.6)	4019.2 (3828.0-4220.0)	.28
Latency of trials with delays, ms^b^	3841.3 (3657.6-4034.1)	4012.1 (3712.9-4335.5)	3700.0 (3476.6-3938.3)	.10
Correct, %	48.3 (46.5-50.1)	47.1 (44.2-49.9)	49.3 (46.9-51.7)	.23
Correct trials with delays, %	39.7 (37.8-41.5)	38.7 (35.9-41.5)	40.5 (38.0-43.1)	.33
Error given correct answer, %	54.7 (52.2-57.1)	54.7 (51.2-58.2)	54.7 (51.1-58.2)	.99
Error given error answer, %	48.4 (45.9-50.8)	5.0 (46.3-53.6)	47.0 (43.7-50.2)	.23

Abbreviations: BLC, Big/Little Circle; DMS, Delayed Matching to Sample; MOT, Motor Screening; SSP, Spatial Span.

^a^ Calculated using a 2-sided *t* test.

^b^ Indicates geometric mean.

The SSP task length ranged from 0 to 5 correct squares tapped within the same sequence, with a mean span length of 2.7 (95% CI, 2.6-2.9) squares. The mean time needed to initiate the SSP assignments was 3666.3 (95% CI, 3498.2-3842.4) milliseconds. Finally, the mean percentage of trials that was answered correctly in the DMS task was 48.3% (95% CI, 46.5%-50.1%) and 39.7% (95% CI, 37.8%-41.5%) when only the assignments in which choice figures were displayed with a delay were taken into account. The percentage of incorrect answers, given that the previous answer was correct, was 54.7% (95% CI, 52.2%-57.1%), and given that the previous answer was incorrect, 48.4% (95% CI, 45.9%-50.8%). The mean time to touch the stimulus on the screen in all DMS trials where the correct stimulus was selected was 4102.2 (95% CI, 3942.5-4268.5) milliseconds, and for only the assignments with delays, 3841.3 (95% CI, 3657.6-4034.1) milliseconds.

### Associations Between Cognitive Functioning and the Retinal Microvasculature

#### Attention and Psychomotor Speed

Principal components for attention and psychomotor speed were derived as linear combinations of the 3 outcomes of the MOT and BLC task. The first principal component explained 49% of the variance; the second, an additional 34%. Characteristics of the principal components used in the multivariable linear regression models are shown in eTable 1 in the Supplement. After adjustment for sex, gestational age, birth weight, ethnicity, BMI, and age, CRVE was negatively associated with the second principal component (*P* = .01). None of the other retinal vessel characteristics was associated with the principal components.

**Figure 2.**
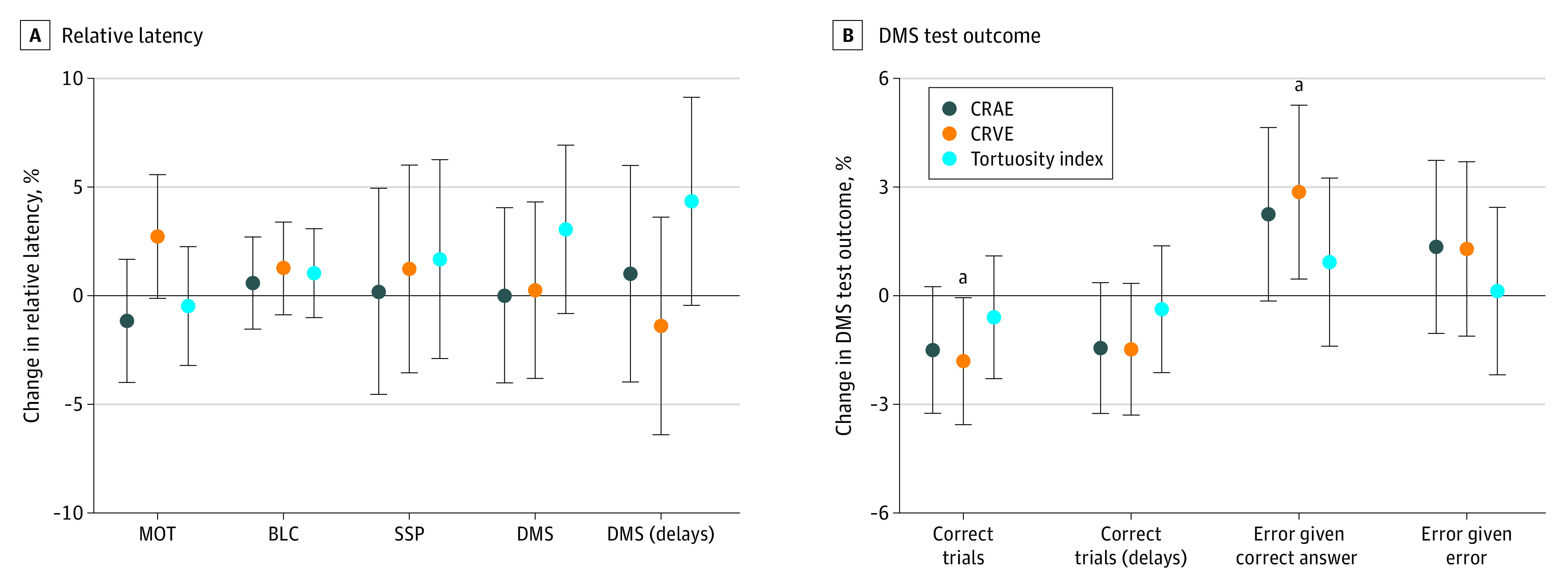
Associations Between the Central Retinal Arterial Equivalent (CRAE) and Central Venular Arteriolar Equivalents (CRVE) and Tortuosity Index and the Mean Latencies Latencies were measured for each of the 4 Cambridge Neuropsychological Test Automated Battery tasks (A) and 3 DMS task outcomes (B). The magnitude of all estimates is expressed for every 1-SD increment in retinal vessel characteristic. Multiple linear regression models were adjusted for age, sex, body mass index, and ethnicity of the child. Abbreviations: BLC, Big/Little Circle task; DMS, Delayed Matching to Sample; MOT, Motor Screening task; and SSP, Spatial Span task. Error bars indicate 95% CIs. ^a^*P* ≤ .05.

With the multiple linear regression analyses for the 3 separate test outcomes, adjusted for the aforementioned variables, a negative association between CRVE and the mean accuracy of the MOT task (−0.39 [95% CI, −0.78 to −0.10] pixel units; *P* = .01) was identified. This was accompanied by a borderline positive association between the CRVE and the reaction time of the MOT test: for every 1-SD increase in CRVE, the children performed 2.74% (95% CI, −0.12% to 5.49%; *P* = .06) slower on the MOT task (Figure 2A). There was no association between any of the retinal vessel characteristics and the reaction speeds recorded in the BLC task (Figure 2A). Further adjustments for MAP, follow-up season, time of CANTAB administration, and problem score decreased the estimated MOT latency for every 1-SD widening in CRVE (1.76%; 95% CI, −1.37% to 5.10%; *P* = .27) but did not substantially alter the other estimates for the MOT and BLC tasks (eTable 2 in the Supplement).

#### Short-Term Memory Assessments: Visuospatial Working Memory and Visual Recognition Memory

In the principal component analysis for short-term memory, the first principal component explained 40% of the variance; the second, an additional 20% to the explained variance of the model (eTable 1 in the Supplement). CRVE was positively associated with the first principal component (*P* = .01) when adjusted for sex, gestational age, birth weight, ethnicity, BMI, and age. No other significant differences were found.

In the multiple linear regression analyses of the separate SSP and DMS task outcomes, vessel tortuosity showed a borderline significant association with DMS latency, when only DMS trials with delays were considered: for every unit increase in tortuosity index, there was a 4.32% (95% CI, −0.48% to 9.12%; *P* = .07) slower performance (Figure 2A). Following additional adjustments for MAP, follow-up season, time of CANTAB administration, and problem score, the latter estimate increased: a 1-unit increase in vessel tortuosity was associated with a 6.48% (95% CI, 0.84%-12.12%; *P* = .02) slower performance in DMS trials with delays and a 4.20% (95% CI, −0.36% to 8.76%; *P* = .07) increase in latency for all DMS trails (eTable 2 in the Supplement). For the DMS task, other assessed parameters than latency showed significant associations with the retinal vessel characteristics. A widening of both vessel types had an effect on the amount of errors given the previous answer being correct: a 1-SD widening of the retinal arteriolar diameter and venular diameter was associated with a 2.30% (95% CI, −0.14% to 4.61; *P* = .07) and 2.94% (95% CI, 0.39% to 5.29%; *P* = .02) increase in the amount of errors, respectively. Moreover, the total percentage of correctly answered trials decreased with 1.44% (95% CI, −3.25% to 0.29%; *P* = .09) for every 1-SD increase in retinal arteriolar diameter and with 1.76% (95% CI, −3.53% to −0.04%; *P* = .04) for a 1-SD increase in retinal venular diameter (Figure 2B). Every SD increase in D_1_ and D_2_ was associated with a 1.71% (95% CI, −0.15% to 3.57%; *P* = .07) and a 1.77% (95% CI, −0.15% to 3.57%; *P* = .05) increase in correctly answered DMS queries, respectively. Additional adjustments did not alter the associations between the DMS outcome parameters and the CRVE (eTable 2 in the Supplement) but increased the estimates for D_1_ and D_2_ to 2.10% (95% CI, 0.06%-4.11%; *P* = .04) and 2.16% (95% CI, 0.21% to 4.11%; *P* = .03) correctly answered DMS tasks, respectively.

### Sensitivity Analyses

We reran our models with additional adjustment for pregnancy-related maternal characteristics, but this did not alter the described associations (eTable 3 in the Supplement). When excluding mothers who consumed alcohol during pregnancy, the latency parameters in association with the retinal venular diameter and the tortuosity increased: the MOT latency increased to 4.12% (95% CI, 0.98% to 7.25%; *P* = .01) for every 1-SD increase in venular diameter, and DMS latency increased to 5.40% (95% CI, −0.24% to 11.04%; *P* = .06) for every 1-SD increase in vessel tortuosity (eTable 4 in the Supplement).

The third sensitivity analysis, excluding children with a higher indication of emotional stress at the time of CANTAB administration, did not alter the association between CRVE and MOT latency, although the association between the tortuosity index and DMS latency lost its significance. This analysis slightly increased the estimates of the percentage of errors made given a previous correct answer and slightly decreased the estimates of the percentage of correct answers of the DMS task, in association with both the retinal arteriolar and venular diameter (eTable 5 in the Supplement). Finally, the fourth sensitivity analysis, excluding children who were born prematurely, did not change the associations between retinal vessel parameters and CANTAB test outcomes (eTable 6 in the Supplement). Only the association between CRVE and MOT latency became significant (3.14%; 95% CI, 0.39%-6.08%; *P* = .03).

## Discussion

The microcirculatory system functions as the exchange surface for oxygen, nutrients, and metabolites between the blood and the parenchymal cells of the body’s tissues. The retinal vessel structure gives a noninvasive view on the microvasculature, and because the retina can be considered a “window to the brain,” it is used as a proxy to study the cerebral microcirculation. Proper functioning and structure of the microvasculature is essential for neurological function and development. In this context, we studied the associations between several domains of neurological development and phenotypes of the retinal microvasculature in children aged 4 to 5 years enrolled in the longitudinal ENVIRONAGE birth cohort. The key findings of this study are that (1) a wider CRVE and an increased vessel tortuosity are associated with a lower short-term visual recognition memory performance; (2) a wider CRVE is associated with a slower performance in the MOT test; and (3) an increased information dimension (D_1_) and correlation dimension (D_2_) are associated with a better performance in short-term memory assessment. This is the first study, to our knowledge, to find an association between retinal vessel characteristics and different areas of neurological development in children as young as 4 years.

Structural changes in the retinal microvasculature have been assessed as potential biomarkers of neurological conditions in adults. For patients with Parkinson disease, the morphology of the retinal veins, rather than the diameter, seems to be affected by the disease process.^18^ Cheung and colleagues^19^ found that persons with Alzheimer disease (mean age of 74.8 years) had narrower retinal venules and an increased vessel tortuosity compared with the matched control population. A large follow-up study in 12 317 participants^20^ in which neurological tests were performed 3 times during a period of 20 years indicated that a narrowing of the retinal arteriolar diameter was associated with a decrease in 20-year cognitive change, although these results did not persist in more elaborate adjusted models. In 244 patients with type 1 diabetes, both a narrower CRAE and wider CRVE were associated with lower scoring on a set of tests assessing mental efficiency and executive function and with a poorer performance for verbal memory for a narrower arteriolar diameter.^21^

Although neurological development in children has been evaluated under several clinical conditions and environmental exposures,^22^ few studies have examined the link between cognitive functioning and retinal vessel characteristics in early life. Recently, Wei and colleagues^10^ compared retinal vessel diameters between preterm (n = 93) and term-born (n = 87) children at 11 years of age. They found an association between narrowing of the retinal arterioles and a poorer performance in matrix reasoning (−1.77 points; *P* = .004) and spatial span length (−2.03 points; *P* = .002) of the Wechsler Non–Verbal Scale of Ability,^10^ the latter in contrast to our findings. A lack of association between SSP test outcomes and retinal vessel measurements in our study can be explained by the age difference at which the tests were administered: even in infants, 5 months compared with 12 months of age can result in a greater number of correct responses in a simplified form of working memory testing.^23^ Moreover, Luciana and Nelson^24^ performed several of the CANTAB tasks in children ranging from ages 4 to 12 years and found significant differences in the performance between these age groups. Therefore, it is crucial that our CANTAB tasks are repeated in other cohorts and during the next follow-up steps in our ENVIRONAGE prospective birth cohort.

We found a slower reaction speed in the MOT test in association with a widening of the retinal venular diameter. The effects of vessel properties on psychomotor speed have mostly been researched in adults and elderly populations. In a study of 77 adults with type 1 diabetes, reduced blood flow in the basal ganglia was associated with slower psychomotor speed.^25^ Kim and colleagues^26^ looked at a combined measure of microvascular abnormalities and found an association between a high level of microvascular burden and reduced scoring on psychomotor assessment.

Visual short-term memory is a complex neurological phenomenon, regulated by an intrinsic cooperation of different areas of the brain. As young as 6 months, this memory system becomes apparent, and it rapidly develops throughout early childhood.^27,28^ A recent follow-up study in 124 children, assessed at 4 and a second time at 7 years of age, revealed that outcomes on visual short-term memory in children aged 4 years were associated with mathematical achievement at 7 years of age (*r* = 0.34; *P* ≤ .01).^29^ Our results suggest that retinal vessel diameter and tortuosity are underlying phenomena relevant in explaining short-term memory, elaborating the current knowledge on the complex process of memory formation and cognitive development.

Neurocognitive processes depend on a proper microvascular architecture, with its functioning intertwined with several factors, such as the endothelial cell activity of the blood-brain barrier.^30,31^ Arteriolar and venular diameters determine adequate exchange of oxygen and nutrients to the peripheral tissues, and these metrics are instrumental for the characterization of the microvasculature. Widening of the arterioles and venules could lower the pressure in these vessels, whereas enough pressure is required for adequate diffusion of oxygen and removal of metabolic waste products.^32^ Reduced supply of oxygen and nutrients has been proven an important cause of poorer results in neurocognitive testing.^33,34^ These observations, combined with our findings, can provide further insight in a pathophysiological model on the effects of microvascular changes on neurocognitive development, initiated during childhood.

Another potential association between retinal vessel changes and cognition could be increased inflammation. Increased levels of inflammatory markers have been associated with changes in retinal vessel diameter^35^ and a decline in cognitive functioning in adults.^36^ A wider retinal venular diameter has been associated with higher levels of C-reactive protein, a marker of inflammation, among children aged 6 years in the Generation R Study^37^ as well as in a German population of school-aged children (mean [SD] age, 11.1 [0.6] years).^38^ Studies in adults have shown that retinopathy^39^ and retinal angular and branching abnormalities^40^ are associated with decreased neurocognitive scoring. Hypothesized is that these abnormalities give rise to increased shear stress in the microvasculature, resulting in increased inflammatory reactions at the endothelial lining of the blood-brain barrier. Future studies should therefore focus on the potential intermediary role of inflammation in the retina-brain connection.

### Strengths and Limitations

A strength of this study is that the retinal image analyses were performed by a single researcher who was blinded for all study conditions, thereby excluding examiner bias. Furthermore, not many normative data of CANTAB tasks are available among children as young as 4 years.^41^ Only Luciana and Nelson^24,42^ tested a large sample of children at this age. With this study, we provide additional normative data on CANTAB test outcomes in a population of healthy children aged 4 to 6 years. A limitation of this study is that performing neurocognitive assessments, such as the CANTAB tasks, in children aged 4 years remains a challenge owing to several age-related factors.^42^ For example, owing to insufficient finger pressure or surface area, some children fail to generate a response at first attempt. However, by implying a test round with several trials before commencing each of the 4 tasks, the children learned how much pressure is needed to register their answer, therefore reducing the chance of not generating enough finger pressure in the registered trials. Moreover, the 4 tasks in the CANTAB battery were determined a priori, taking into consideration the young age of the participants, and by using a standardized script for test administration the proper execution of the tests is directed.

## Conclusions

In this cohort study, a widening of the retinal venular diameter and increased information dimension (D_1_) and correlation dimension (D_2_) were associated with the outcome of established neurodevelopmental tests and can be regarded as potential biomarkers to evaluate neurobehavioral development in children as young as 4 years. These noninvasive retinal measurements can support investigations into microvascular changes that accompany neurological development at a young age. Neurological assessments and retinal imaging at future follow-up visits of the ENVIRONAGE prospective birth cohort will further elaborate these findings.

## Notes

Supplement. **eFigure.** Flowchart Summarizing the Selection Process Resulting in the 251 Participants of This Study **eTable 1.** Characteristics of the First and Second Principal Components **eTable 2.** Associations of the Multiple Linear Regression Analyses Between Retinal Vessel Characteristics and Outcomes of the 4 CANTAB Tasks **eTable 3.** Sensitivity Analysis Additionally Adjusting the Multiple Linear Regression Model Between Retinal Vessel Characteristics and Outcomes of the 4 CANTAB Tasks **eTable 4.** Sensitivity Analysis Excluding Mothers Who Consumed Alcohol During Pregnancy (n = 50) **eTable 5.** Sensitivity Analysis Excluding Children With a Problem Score Between 14 and 40 Points (n = 33) **eTable 6.** Sensitivity Analysis Excluding Children Who Were Born Preterm (Gestational Age < 38 wk) (n = 11)
