# Supplementary material for: Association of Retinal Microvascular Characteristics With Short-term Memory Performance in Children Aged 4 to 5 Years
Source: JAMA Netw Open. 2020 Jul 24;3(7):e2011537. doi: 10.1001/jamanetworkopen.2020.11537 (PMC7382002; doi:10.1001/jamanetworkopen.2020.11537)
Supplement: Supplement. — eFigure. Flowchart Summarizing the Selection Process Resulting in the 251 Participants of This Study eTable 1. Characteristics of the First and Second Principal Components eTable 2. Associations of the Multiple Linear Regression Analyses Between Retinal Vessel Characteristics and Outcomes of the 4 CANTAB Tasks eTable 3. Sensitivity Analysis Additionally Adjusting the Multiple Linear Regression Model Between Retinal Vessel Characteristics and Outcomes of the 4 CANTAB Tasks eTable 4. Sensitivity Analysis Excluding Mothers Who Consumed Alcohol During Pregnancy (n = 50) eTable 5. Sensitivity Analysis Excluding Children With a Problem Score Between 14 and 40 Points (n = 33) eTable 6. Sensitivity Analysis Excluding Children Who Were Born Preterm (Gestational Age < 38 wk) (n = 11) [file jamanetwopen-3-e2011537-s001.pdf]

Supplementary Online Content

Luyten LJ, Dockx Y, Madhloum N, et al. Association of retinal microvascular characteristics with short-term memory performance in children aged 4 to 5 years. *JAMA Netw Open*. 2020;3(7):e2011537. doi:10.1001/jamanetworkopen.2020.11537

eFigure. Flowchart Summarizing the Selection Process Resulting in the 251 Participants of This Study

eTable 1. Characteristics of the First and Second Principal Components

eTable 2. Associations of the Multiple Linear Regression Analyses Between Retinal Vessel Characteristics and Outcomes of the 4 CANTAB Tasks

eTable 3. Sensitivity Analysis Additionally Adjusting the Multiple Linear Regression Model Between Retinal Vessel Characteristics and Outcomes of the 4 CANTAB Tasks

eTable 4. Sensitivity Analysis Excluding Mothers Who Consumed Alcohol During Pregnancy (n = 50)

eTable 5. Sensitivity Analysis Excluding Children With a Problem Score Between 14 and 40 Points (n = 33)

eTable 6. Sensitivity Analysis Excluding Children Who Were Born Preterm (Gestational Age < 38 wk) (n = 11)

This supplementary material has been provided by the authors to give readers additional information about their work.

©2020 Luyten LJ et al. *JAMA Network Open*.

eFigure. Flowchart Summarizing the Selection Process Resulting in the 251 Participants of This Study

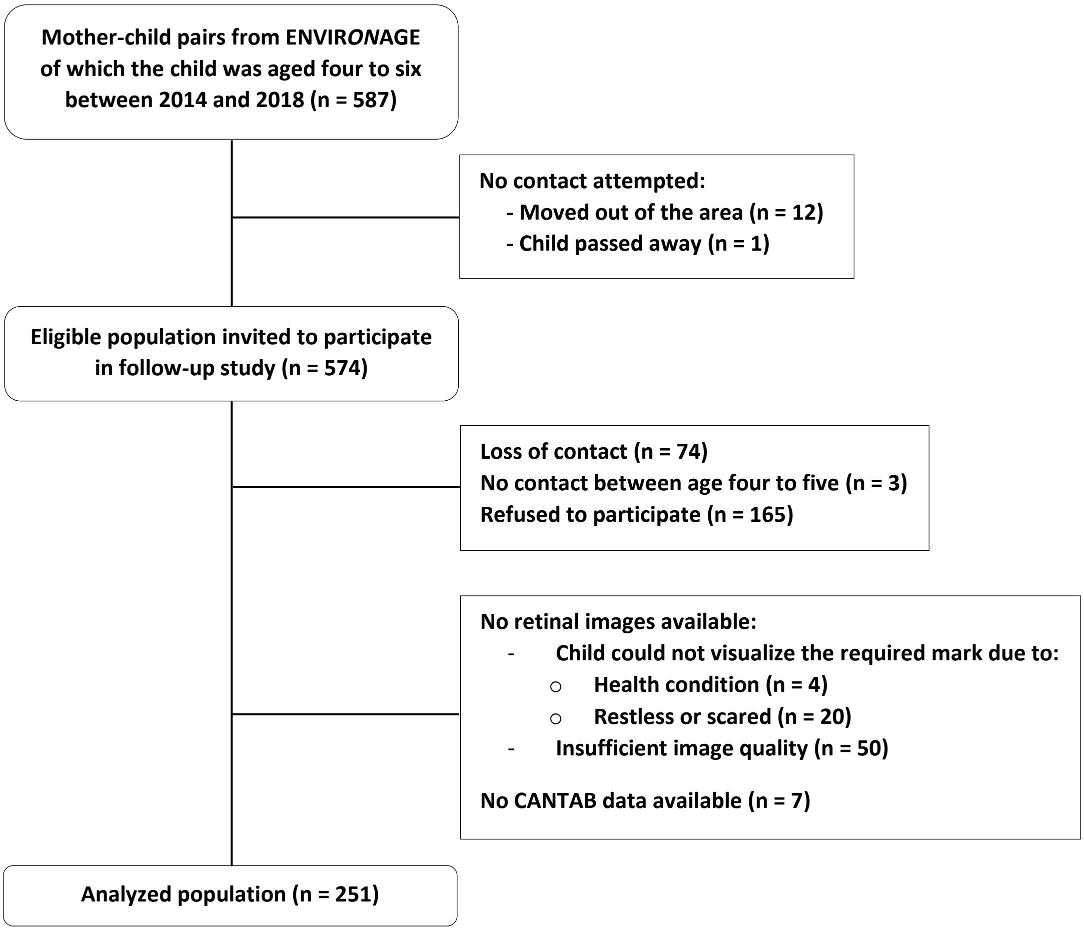

©2020 Luyten LJ et al. *JAMA Network Open*.

eTable 1. Characteristics of the First and Second Principal Components

Neurocognitive domain	Eigenvalue PC1 [Explained variance]	Eigenvalue PC2 [Explained variance]	CANTAB test outcome	Eigenvector PC1	Eigenvector PC2
Attention and psychomotor speed	1.47 [49%]	1.00 [34%]	MOT latency	0.71	0.06
			MOT mean error	0.06	0.99
			BLC latency	0.70	-0.14
Short term memory	2.40 [40%]	1.20 [20%]	SSP span	-0.24	-0.39
			SSP latency first response	0.09	0.68
			DMS latency	-0.09	0.60
			DMS percentage correct	-0.63	0.07
			DMS error given correct	0.50	-0.14
			DMS error given error	0.53	0.03

Abbreviations: BLC, Big/Little Circle; DMS, Delayed matching to sample; MOT, Motor screening task; SSP, Spatial Span.

©2020 Luyten LJ et al. *JAMA Network Open*.

eTable 2. Associations of the Multiple Linear Regression Analyses Between Retinal Vessel Characteristics and Outcomes of the 4 CANTAB Tasks

CANTAB test and outcome(s)	CRAE	CRVE	TI
MOT			
Mean latency	-1.01 (-4.32; 2.30)	1.76 (-1.37; 5.10)	-0.84 (-3.96; 2.28)
Mean accuracy	-0.14 (-0.58; 0.14)	-0.78 (-1.18; -0.39)*	0.12 (-0.24; 0.60)
BLC			
Mean latency	1.15 (-1.44; 3.74)	1.37 (-1.18; 3.92)	1.44 (-0.96; 3.84)
SSP			
Span length	-0.06 (-0.29; 0.12)	-0.08 (-0.20; 0.10)	-0.02 (-0.24; 0.12)
Mean latency of first response	1.73 (-4.03; 7.49)	1.76 (-3.92; 7.25)	3.24 (-2.04; 8.64)
DMS			
Mean latency	1.73 (-3.17; 6.62)	1.57 (-3.33; 6.27)	4.20 (-0.36; 8.76)
Mean latency (trials with delays)	2.45 (-3.60; 8.50)	-0.39 (-6.27; 5.49)	6.48 (0.84; 12.12)*
Error given correct	1.44 (-1.30; 4.32)	2.74 (-0.08; 5.29)	0.36 (-2.28; 3.00)
Error given error	1.58 (-1.30; 4.32)	1.76 (-0.98; 4.51)	0.24 (-2.40; 2.88)
Percentage Correct	-1.44 (-3.46; 0.72)	-1.76 (-3.92; 0.18)	-0.24 (-2.16; 1.80)
Percentage Correct (trials with delays)	-1.58 (-3.74; 0.58)	-1.57 (-3.72; 0.59)	-0.24 (-2.28; 1.80)

Estimated changes (% change and 95% CI) in CANTAB test outcomes are given for every 1-SD increase in CRAE, CRVE, or TI. Models were adjusted for age, sex, ethnicity, BMI, MAP, problem score, time at which the CANTAB test battery was initiated and season during which the follow-up visit took place. Complete data were available for 208 mother-child pairs. Abbreviations: BLC, Big/Little Circle; CRAE, Central retinal arteriolar equivalent; CRVE, Central retinal venular equivalent; DMS, Delayed matching to sample; MOT, Motor screening task; SSP, Spatial Span; TI, Tortuosity index. *p ≤ 0.05.

©2020 Luyten LJ et al. *JAMA Network Open*.

eTable 3. Sensitivity Analysis Additionally Adjusting the Multiple Linear Regression Model Between Retinal Vessel Characteristics and Outcomes of the 4 CANTAB Tasks

CANTAB test and outcome(s)	CRAE	CRVE	TI
MOT			
Mean latency	-0.29 (-3.74; 3.02)	2.16 (-1.18; 5.49)	-2.52 (-5.88; 0.84)
Mean accuracy	-0.14 (-0.58; 0.29)	-0.59 (-0.98; -0.18)*	0.24 (-0.24; 0.60)
BLC			
Mean latency	0.14 (-2.45; 2.88)	0.59 (-2.16; 3.14)	1.80 (-0.84; 4.44)
SSP			
Span length	-0.06 (-0.29; 0.13)	-0.14 (-0.39; 0.06)	-0.02 (-0.24; 0.12)
Mean latency of first response	3.31 (-2.88; 9.50)	3.14 (-2.94; 9.21)	3.12 (-3.00; 9.36)
DMS			
Mean latency	0.72 (-4.46; 5.90)	0.59 (-4.51; 5.68)	6.48 (1.44; 11.52)*
Mean latency (trials with delays)	1.58 (-4.61; 7.92)	-1.37 (-7.45; 4.90)	8.04 (1.80; 14.16)*
Error given correct	1.44 (-1.58; 4.46)	2.35 (-0.59; 5.49)	-0.24 (-3.36; 2.76)
Error given error	1.73 (-1.15; 4.61)	1.96 (-0.98; 4.70)	-0.60 (-3.36; 2.28)
Percentage Correct	-1.44 (-3.60; 0.72)	-1.76 (-3.92; 0.39)	0.60 (-1.68; 2.76)
Percentage Correct (trials with delays)	-1.73 (-4.03; 0.58)	-1.57 (-3.92; 0.78)	-0.36 (-1.92; 2.64)

Estimated changes (% change and 95% CI) in CANTAB test outcomes are given for every 1-SD increase in CRAE, CRVE, or TI. Models were adjusted for age, sex, ethnicity, BMI, MAP, problem score, time at which the CANTAB test battery was initiated, season during which the follow-up visit took place, education level of both parents, maternal smoking and alcohol consumption during pregnancy and exposure of the child to passive smoking in its household environment. Education level was coded as low (no diploma or primary school), middle (high school diploma), or high (college or university degree). Maternal smoking status during pregnancy was defined as non-smokers, stopped smoking before pregnancy, and current smokers (smoked during pregnancy). Alcohol use of the mother during pregnancy was subdivided in mothers who did not consume alcohol during pregnancy, and mothers who consumed alcohol at least occasionally during gestation. Complete data were available for 188 participants. Abbreviations: BLC, Big/Little Circle; CRAE, Central retinal arteriolar equivalent; CRVE, Central retinal venular equivalent; DMS, Delayed matching to sample; MOT, Motor screening task; SSP, Spatial Span; TI, Tortuosity index. *p ≤ 0.05.

©2020 Luyten LJ et al. *JAMA Network Open*.

eTable 4. Sensitivity Analysis Excluding Mothers Who Consumed Alcohol During Pregnancy (n = 50)

CANTAB test and outcome(s)	CRAE	CRVE	TI
MOT			
Mean latency	-1.01 (-4.18; 2.16)	4.12 (0.98; 7.25)*	0.24 (-3.12; 3.60)
Mean accuracy	-0.13 (-0.58; 0.29)	-0.39 (-0.78;-0.06)*	-0.08 (-0.48; 0.36)
BLC			
Mean latency	0.86 (-1.58; 3.31)	1.18 (-1.18; 3.53)	1.08 (-1.32; 3.60)
SSP			
Span length	0.04 (-0.12; 0.14)	-0.02 (-0.18; 0.14)	-0.01 (-0.12; 0.12)
Mean latency of first response	-0.10 (-5.47; 5.18)	1.18 (-4.12; 6.47)	2.40 (-3.00; 7.92)
DMS			
Mean latency	-0.01 (-4.61; 4.61)	-0.39 (-4.90; 4.31)	3.24 (-1.44; 7.92)
Mean latency (trials with delays)	1.87 (-3.74; 7.34)	-0.98 (-6.47; 4.51)	5.04 (-0.60; 10.80)
Error given correct	1.01 (-1.73; 3.60)	2.35 (-0.18; 4.90)	1.08 (-1.56; 3.84)
Error given error	1.15 (-1.73; 3.89)	1.37 (-1.37; 4.12)	-0.48 (-3.36; 2.40)
Percentage Correct	-0.86 (-2.74; 1.15)	-1.76 (-3.53; 0.20)	-0.60 (-2.64; 1.32)
Percentage Correct (trials with delays)	-0.72 (-2.74; 1.15)	-1.18 (-3.14; 0.78)	-0.48 (-2.52; 1.56)

Estimated changes (% change and 95% CI) in CANTAB test outcomes are given for every 1-SD increase in CRAE, CRVE, or TI. Models were adjusted for age, sex, ethnicity, and BMI. Abbreviations: BLC, Big/Little Circle; CRAE, Central retinal arteriolar equivalent; CRVE, Central retinal venular equivalent; DMS, Delayed matching to sample; MOT, Motor screening task; SSP, Spatial Span; TI, Tortuosity index. *p ≤ 0.05

©2020 Luyten LJ et al. *JAMA Network Open*.

eTable 5. Sensitivity Analysis Excluding Children With a Problem Score Between 14 and 40 Points (n = 33)

CANTAB test and outcome(s)	CRAE	CRVE	TI
MOT			
Mean latency	-0.58 (-3.60; 2.45)	2.55 (-0.59; 5.68)	-0.36 (-3.24; 2.52)
Mean accuracy	-0.07 (-0.43; 0.29)	-0.39 (-0.78; 0.02)	0.07 (-0.24; 0.48)
BLC			
Mean latency	1.15 (-1.01; 3.31)	1.57 (-0.59; 3.72)	1.32 (-0.72; 3.48)
SSP			
Span length	0.06 (-0.10; 0.14)	-0.04 (-0.20; 0.10)	-0.01 (-0.12; 0.12)
Mean latency of first response	-0.09 (-5.04; 4.90)	1.37 (-3.72; 6.27)	0.96 (-3.96; 5.76)
DMS			
Mean latency	0.43 (-3.74; 4.61)	0.39 (-3.72; 4.70)	3.24 (-0.72; 7.32)
Mean latency (trials with delays)	1.01 (-4.03; 6.05)	-1.37 (-6.47; 3.72)	3.48 (-1.32; 8.40)
Error given correct	3.02 (0.43; 5.62)*	3.53 (0.98; 6.08)*	0.48 (-2.04; 3.00)
Error given error	1.44 (-1.01; 4.03)	1.57 (-1.18; 4.12)	-0.48 (-2.88; 2.04)
Percentage Correct	-1.87 (-3.74; 0.04)*	-2.16 (-4.12; -0.39)*	-0.05 (-1.92; 1.80)
Percentage Correct (trials with delays)	-1.58 (-3.60; 0.43)	-1.76 (-3.72; 0.12)	0.12 (-1.68; 2.04)

Estimated changes (% change and 95% CI) in CANTAB test outcomes are given for every 1-SD increase in CRAE, CRVE, or TI. Models were adjusted for age, sex, ethnicity, and BMI. Abbreviations: BLC, Big/Little Circle; CRAE, Central retinal arteriolar equivalent; CRVE, Central retinal venular equivalent; DMS, Delayed matching to sample; MOT, Motor screening task; SSP, Spatial Span; TI, Tortuosity index. *p ≤ 0.05

©2020 Luyten LJ et al. *JAMA Network Open*.

eTable 6. Sensitivity Analysis Excluding Children Who Were Born Preterm (Gestational Age < 38 wk) (n = 11)

CANTAB test and outcome(s)	CRAE	CRVE	TI
MOT			
Mean latency	-1.30 (-4.18; 1.44)	3.14 (0.39; 6.08)*	-0.48 (-3.36; 2.28)
Mean accuracy	-0.09 (-0.43; 0.29)	-0.39 (-0.78; -0.08)*	-0.02 (-0.36; 0.36)
BLC			
Mean latency	0.43 (-1.73; 2.59)	1.18 (-0.98; 3.33)	1.08 (-1.08; 3.24)
SSP			
Span length	0.06 (-0.09; 0.14)	-0.04 (-0.18; 0.12)	-0.02 (-0.24; 0.12)
Mean latency of first response ms)	0.13 (-4.61; 4.90)	1.18 (-3.72; 6.08)	0.48 (-4.08; 5.16)
DMS			
Mean latency	-0.03 (-4.18; 4.03)	-0.06 (-4.31; 4.12)	3.12 (-0.96; 7.08)
Mean latency (trials with delays)	0.86 (-4.18; 6.05)	-1.76 (-6.86; 3.33)	4.32 (-0.60; 9.36)
Error given correct	2.02 (0.29; 4.46)	3.14 (0.59; 5.49)*	1.20 (-1.20; 3.48)
Error given error	1.30 (-1.15; 3.74)	1.18 (-1.37; 3.53)	0.24 (-2.16; 2.64)
Percentage Correct	-1.30 (-3.17; 0.43)	-1.76 (-3.53; 0.02)*	-0.72 (-2.52; 0.96)
Percentage Correct (trials with delays)	-1.44 (-3.17; 0.43)	-1.57 (-3.33; 0.39)	-0.36 (-2.28; 1.32) 7)

Estimated changes (% change and 95% CI) in CANTAB test outcomes are given for every 1-SD increase in CRAE, CRVE, or TI. Models were adjusted for age, sex, ethnicity, and BMI. Abbreviations: BLC, Big/Little Circle; CRAE, Central retinal arteriolar equivalent; CRVE, Central retinal venular equivalent; DMS, Delayed matching to sample; MOT, Motor screening task; SSP, Spatial Span; TI, Tortuosity index. *p ≤ 0.05

©2020 Luyten LJ et al. *JAMA Network Open*.
